# Arsenic Trioxide Induces Apoptosis in Human Platelets via C-Jun NH_2_-Terminal Kinase Activation

**DOI:** 10.1371/journal.pone.0086445

**Published:** 2014-01-22

**Authors:** Yicun Wu, Jin Dai, Weilin Zhang, Rong Yan, Yiwen Zhang, Changgeng Ruan, Kesheng Dai

**Affiliations:** 1 Jiangsu Institute of Hematology, The First Affiliated Hospital of Soochow University, Key Laboratory of Thrombosis and Hemostasis, Ministry of Health, Suzhou, China; 2 School of Life Sciences, Peking University, Beijing, China; Chang Gung University, Taiwan

## Abstract

Arsenic trioxide (ATO), one of the oldest drugs in both Western and traditional Chinese medicine, has become an effective anticancer drug, especially in the treatment of acute promyelocytic leukemia (APL). However, thrombocytopenia occurred in most of ATO-treated patients with APL or other malignant diseases, and the pathogenesis remains unclear. Here we show that ATO dose-dependently induces depolarization of mitochondrial inner transmembrane potential (ΔΨm), up-regulation of Bax and down-regulation of Bcl-2 and Bcl-X_L_, caspase-3 activation, and phosphotidylserine (PS) exposure in platelets. ATO did not induce surface expression of P-selectin and PAC-1 binding, whereas, obviously reduced collagen, ADP, and thrombin induced platelet aggregation. ATO dose-dependently induced c-Jun NH_2_-terminal kinase (JNK) activation, and JNK specific inhibitor dicumarol obviously reduced ATO-induced ΔΨm depolarization in platelets. Clinical therapeutic dosage of ATO was intraperitoneally injected into C57 mice, and the numbers of circulating platelets were significantly reduced after five days of continuous injection. The data demonstrate that ATO induces caspase-dependent apoptosis via JNK activation in platelets. ATO does not incur platelet activation, whereas, it not only impairs platelet function but also reduces circulating platelets *in vivo*, suggesting the possible pathogenesis of thrombocytopenia in patients treated with ATO.

## Introduction

Arsenic trioxide (ATO), one of the oldest drugs in both Western and traditional Chinese medicine, has become one of the most effective anticancer drugs, especially in the treatment of acute promyelocytic leukemia (APL) [1]. ATO appears to be the most effective single agent in the treatment of APL and there have been very few reports of primary resistance [2]. Furthermore, ATO has been approved by the Federal Drug Administration for treating all-trans retinoic acid (ATRA)-resistant APL [3]. In addition, ATO has appeared to be one of the most promising general anticancer drugs for many kinds of malignance, such as lymphoma [4], hepatocellular carcinoma [5], myelodysplastic syndrome [6]. However, the same as other anticancer drugs, there are many adverse events associated with ATO treatment including thrombocytopenia and hemorrhage which are also the main cause of death in some prime diagnosis or under treatment APL patients [7], [8]. Thrombocytopenia occurred in most of the relapsed or refractory APL patients treated by ATO [9], and durations of thrombocytopenia were significantly longer during ATO therapy compared with ATRA [10]. Furthermore, studies by different groups also indicate that thrombocytopenia is a frequent hematological side effect during the treatment of ATO in patients with myelodysplastic syndrome [6], multiple myeloma [11], metastatic melanoma [12], and hepatocellular carcinoma [13]. The mechanism, however, is still unclear.

It has been generally accepted that the anticancer effects of ATO via it induces malignant cell apoptosis. ATO induces apoptosis mainly through activating the mitochondria-mediated intrinsic apoptotic pathway [14], [15]. Down-regulation of Bcl-2 gene expression and caspase activation are the indispensable apoptotic processes in ATO-treated malignant or non-malignant cells [16]. Sustained activation of c-Jun NH_2_-terminal kinase (JNK) has been reported to play key roles in ATO-induced apoptosis in various types of cells [17], [18]. Although the phosphoinositide 3-kinase/Akt, NF-kappa B inactivation, XIAP down regulation, and PTEN up regulation signals were reported to involve in ATO-induced apoptosis, ATO-induced JNK activation was crucial and required for subsequent apoptotic events, as inhibition of JNK activity prevented Akt and NF-kappa B inactivation, caspase activation, and mitochondrial damage in B-chronic lymphocytic leukemia cell [18]. However, while a large body of information has depicted the signaling cascades leading to apoptosis in different cell types, it is still unclear whether ATO incurs platelet apoptosis.

Platelet apoptosis induced by either physiological or chemical compounds occurs widely *in vitro* or *in vivo*, which might play important roles in controlling the number of circulating platelets or in the development of platelet-related diseases [19]–[21]. We reported recently that an anticancer drug cisplatin incurs platelet apoptosis [21]. In the current observation, the data demonstrate that ATO induces mitochondria-mediated intrinsic apoptosis of platelets. ATO does not incur platelet activation, whereas, it not only impairs platelet function but also reduces circulating platelets *in vivo*, suggesting the possible pathogenesis of thrombocytopenia in patients treated with ATO.

## Materials and Methods

### Ethics Statement

For studies involving human subjects, approval was obtained from the Soochow University review board. Written informed consent was provided and studies were performed in accordance with the Declaration of Helsinki. Permission for the animal experiment was granted by the Ethics Committee of Laboratory Animals of Soochow University. The proper housing, feeding and care, and all interventions relating to the animal welfare were carried out in compliance with the stipulations of Regulations for the Administration of Affairs Concerning Experimental Animals (China).

### Antibodies and Reagents

Monoclonal antibody SZ51 against P-selectin was from Prof. Changgeng Ruan (Soochow University, Suzhou, China). Sodium hydroxide, dimethyl sulfoxide (DMSO), sodium chloride, aprotinin, dicumarol, ADP, thrombin, ethylenediamine tetra acetic acid-potassium (EDTA-K_2_) and fluorescein isothiocyanate (FITC)-conjugated PAC-1 were purchased from Sigma (St. Louis, Missouri, USA). ATO (As_2_O_3_) was purchased from Beijing Shuanglu Pharmaceutical Co., Ltd. (Beijing, China). FITC-annexin V was purchased from Jiamay Biotech (Beijing, China). FITC-conjugated goat anti-mouse IgG (FITC-GAM) was purchased from Bioworld Technology CO., Ltd. (Minneapolis, USA). Goat anti-mouse immunoglobulin (IgG) conjugated with horseradish peroxidase (GAM-HRP), Goat anti-rabbit IgG conjugated with horseradish peroxidase (GAR-HRP), normal mouse IgG and antibodies against Bax, Bcl-X_L_, Bcl-2, and caspase-3 were purchased from Santa Cruz Biotechnology (Santa Cruz, CA, USA). Anti-GAPDH (glyceraldehyde-3-phosphate dehydrogenase), anti-JNK 1/2, anti-phospho-JNK 1/2 antibodies and 5,5′,6,6′-tetrachloro-1,1′,3,3′-tetraethylbenzimidazolcarbocyanine iodide (jc-1), enhanced chemiluminesence (ECL), phenylmethanesulfonyl fluoride (PMSF) were purchased from Beyotime Institute of Biotechnology (Beyotime, Haimen, China). L-trans-Epoxysuccinyl-leucylamido (4-guanidino) butane (E64) was purchased from Roche Molecular Biochemicals (Indianapolis, IN, USA). Collagen was purchased from Chrono-Log Corp. (Havertown, PA, USA). A23187 was purchased from Calbiochem (San Diego, CA, USA).

### Preparation of Washed Platelets and Platelet-rich Plasma (PRP)

Fresh blood from healthy volunteers was anti-coagulated with 1/7 volume of acid-citrate dextrose (ACD, 2.5% trisodium citrate, 2.0% D-glucose, 1.5% citric acid). After centrifugation, isolated platelets were washed twice with CGS buffer (123 mM NaCl, 33 mM D-glucose, 13 mM trisodium citrate, pH 6.5) and resuspended in modified Tyrode′s buffer (2.5 mM Hepes, 150 mM NaCl, 2.5 mM KCl, 12 mM NaHCO_3_, 1 mM CaCl_2_, 1 mM MgCl_2_, 5.5 mM D-glucose, pH 7.4) to a final concentration of 3×10^8^/mL. For the preparation of PRP, blood was anti-coagulated with 1/9 volume of 3.8% trisodium citrate. After centrifuged at 150×*g* for 12 minutes (min) at room temperature (RT), PRP was isolated. Washed platelets and PRP were then incubated at RT for 1 hour (hr) to recover to resting state as described previously [22], [23].

### Platelet Aggregation Assay

PRP was incubated with ATO (2 μM) or vehicle control (DMSO) at 37°C for 1 hr, washed platelets were incubated with ATO (16 μM) or vehicle control (DMSO) at 37°C for 2 hrs. Platelet aggregation assay was performed by addition of collagen (5 μg/mL) or ADP (10 μmol/L) into PRP, or thrombin (0.5 U/mL) into washed platelets at 37°C, and examined by a turbidometric platelet aggregometer (Chrono-log, PA, USA) at a stirring speed of 1000 rpm [22], [23]. The final concentration of DMSO in each sample did not exceed 0.1%.

### Mitochondrial Inner Transmembrane Potential (Δψm) Depolarization Assay

Washed platelets (3×10^8^/mL) were pre-treated with ATO (2 μM, 4 μM, 8 μM, 16 μM) or vehicle at 37°C for 5 hrs, and then ΔΨm was detected using the lipophilic cationic probe JC-1. JC-1 was added to the pre-treated platelets to a final concentration of 5 μg/mL and then incubated at 37°C in the dark for 20 min. The treated samples were detected by flow cytometry. The JC-1 monomers (λex 514 nm, λem 529 nm) and aggregates (λex 585 nm, λem 590 nm) were calculated as the fluorescence ratio of red (aggregates) to green (monomers). Red fluorescence represents potential-dependent aggregation in the mitochondria, green fluorescence reflects the monomeric form of JC-1 appeared in the cytosol after mitochondrial membrane potential depolarization [24]. In some experiments, platelets were pre-treated with dicumarol (dissolved in weak alkaline solution, 2 μM) at RT for 15 min, and then incubated with ATO (16 μM) or vehicle control at 37°C for 5 hrs, and then ΔΨm was detected with JC-1 by flow cytometry.

### Phosphotidylserine (PS) Exposure Assay

Washed platelets (3×10^8^/mL) were incubated with different concentrations of ATO (2 μM, 4 μM, 8 μM, 16 μM) at 37°C for 5 hrs. Annexin V binding buffer was then mixed with pre-treated platelets and FITC-annexin V at a ratio of 50: 10: 1. Samples were mixed gently and incubated at RT for 15 min in the dark and then subjected to flow cytometry [19].

### Platelet Surface Staining

Washed platelets (3×10^8^/mL) were incubated with different concentrations of ATO (2 μM, 4 μM, 8 μM, 16 μM) or vehicle at 37°C for 5 hrs. For P-selectin surface staining assay, the treated platelets were incubated with SZ51 at RT for 30 min, and then incubated with FITC-GAM in the dark at RT for 30 min and subjected to flow cytometry analysis. In PAC-1 binding assay, platelets were incubated with ATO (2 μM, 4 μM, 8 μM, 16 μM) and then further treated with FITC-labeled soluble PAC-1 and incubated at RT for 20 min in the dark. The treated platelets were fixed with 1% paraformaldehyde, further incubated at 4°C in the dark for 30 min. Then the treated samples were subjected to flow cytometry detection [22]. A23187-treated platelets were set as positive controls, platelets treated with mouse IgG and then incubated with FITC-GAM were set as negative controls.

### Western Blot Analysis

Washed platelets (3×10^8^/mL) were incubated with ATO (2 μM, 4 μM, 8 μM) or vehicle at 37°C for 5 hrs, and then lysed in an equal volume of 2× cell lysis buffer containing 1/100 aprotinin, 1 mM PMSF and 0.1 mM E64 on ice for 30 min, and then subjected to sodium dodecyl sulfate polyacrylamide gel electrophoresis (SDS-PAGE) and Western blot analysis with anti-Bax, anti-Bcl-2, anti-Bcl-X_L_, anti-caspase-3, anti-JNK 1/2, anti-phospho-JNK 1/2, respectively. Anti-GAPDH antibody was used as a loading control to ensure that protein inputs in each group were similar.

### Animal Model

C57BL/6j mice (8–9 weeks) were purchased from Shanghai Institute for Biological Sciences (China). Mice were housed individually under routine conditions at animal facilities of Soochow University at 22–24°C with day-night light cycle of 12 hrs. All interventions relating to the animal welfare were carried out in strict compliance with the stipulations of Regulations for the Administration of Affairs Concerning Experimental Animals (China). After quarantined for two weeks, the mice were randomly allocated into two groups, and the number of male and female mice in the two groups was kept consistent. In ATO group, the mice were injected intraperitoneally with ATO (dissolved in 0.9% normal saline (NS)) at a dose of 5 mg/kg each day for 5 continuous days. In control group, the mice were injected intraperitoneally with the equivalent volume of 0.9% NS.

### Platelet Count

The mice were marked and blood samples of the same mouse were collected from the tip of tail vein after incubated with warm water (37°C) for 3 to 4 min. The first drop of blood was discarded, and 20 μL blood was then collected and anti-coagulated with EDTA-K_2_. Then, local pressing hemostasis was immediately taken to prevent further bleeding [25]. Blood samples were taken before injection and 24 hrs after the final injection. Then, the blood samples were subjected to platelet count assay using Sysmex KX-21N Blood Cell Analyser (Sysmex Corporation, Kobe, Japan).

### Statistical Analysis

Data from more than three sets of specimens were used for statistical analysis. Data are shown as means ± standard deviation (SD). The statistical difference between groups was determined by the paired Student’s *t*-test. A *P*-value less than 0.05 was considered significant.

## Results

### ATO Dose-dependently Induces ΔΨm Depolarization

**Figure 1:**
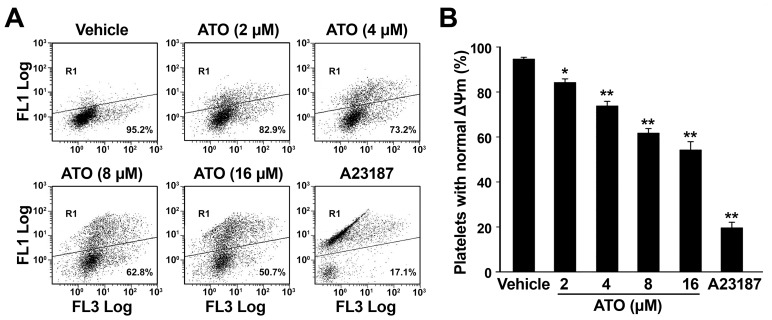
ATO dose-dependently induces ΔΨm depolarization in platelets. Washed platelets were pre-treated with 2, 4, 8, or 16 μM of ATO or vehicle at 37°C for 5 hrs. JC-1 was added into the pre-treated platelets to a final concentration of 5 μg/mL and then incubated at 37°C in the dark for 20 min. Then the treated samples were detected using flow cytometry. (A) Typical flow cytometric histograms represented of 3 separate experiments are shown. FL1 and FL3 stand for green and red fluorescence, respectively. Dots inside R1 indicate that platelets are out of the main population, bearing ΔΨm depolarization. (B) Quantitation data from 3 separate experiments with different donors are illustrated (mean ± SD). *P<0.05, **P<0.01, compared with vehicle controls.

It has been confirmed that ATO induces apoptosis mainly through activating the mitochondria-mediated intrinsic apoptotic pathway in nucleated cells [14], [15], and most of the current reported platelet apoptosis is mitochondrial pathway-dependent [19]–[22]. In order to investigate whether ATO induces platelet apoptosis, platelets were incubated with different concentrations of ATO, and then platelet ΔΨm depolarization was tested by flow cytometry. As shown in Fig. 1, ATO dose-dependently induced ΔΨm depolarization in platelets.

### ATO Induces Up-regulation of Bax, Down-regulation of Bcl-2 and Bcl-X_L_

**Figure 2:**
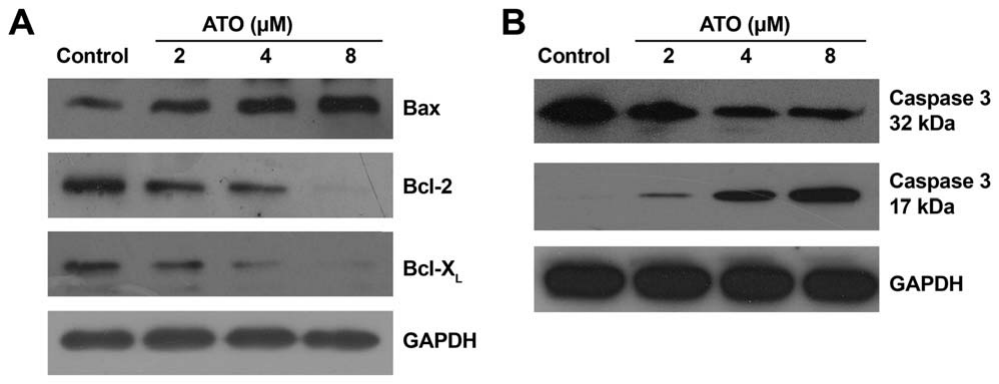
ATO dose-dependently induces up-regulation of Bax, down-regulation of Bcl-2 and Bcl-X_L_, and caspase-3 activation in platelets. Platelets were incubated with 2, 4, or 8 μM of ATO or vehicle at 37°C for 5 hrs. (A) The treated platelets were subjected to Western blot analysis with anti-Bax, anti-Bcl-2, and anti-Bcl-X_L_ antibodies. (B) Pre-treated platelets were subjected to Western blot analysis using anti-caspase-3 antibody. The 32 kDa caspase-3 fragment indicates nonactivated caspase-3, the 17 kDa caspase-3 fragment indicates activated caspase-3. GAPDH levels demonstrate similar loading. Results are representative of 3 separate experiments with different donors.

Bcl-2 family proteins, including anti-apoptotic proteins (e.g. Bcl-2, Bcl-X_L_) and pro-apoptotic proteins (e.g. Bax), interact with the mitochondrial outer membrane, regulating ΔΨm depolarization which is crucial for mitochondria-mediated intrinsic apoptotic pathway [19]–[22]. Therefore, the disequilibrium expression of Bcl-2 family proteins initiates pro-apoptotic or anti-apoptotic signaling cascades. To clarify whether ATO induces platelet apoptotic events through the mitochondrial-dependent pathway, the levels of Bcl-2 family members were examined in platelets pre-incubated with various concentrations of ATO. As shown in Fig. 2A, ATO induced up-regulation of Bax and down-regulation of anti-apoptotic proteins Bcl-2 and Bcl-X_L_ in platelets in a dose-dependent manner, suggesting that ATO induces mitochondrial pathway-dependent platelet apoptosis.

### Caspase-3 is Activated in Platelets Treated with ATO

In nucleate cells, pro-apoptotic Bcl-2 family proteins interact with the mitochondrial outer membrane which leads to the release of cytochrome c, resulting in the activation of caspases, especially caspase-3, the executor of apoptosis [26]. Thus, to further investigate whether ATO induces platelet apoptosis, caspase-3 activation was examined in ATO-treated platelets. Compared with the vehicle control, the 17 kDa caspase-3 fragment, which indicates the activation of caspase-3, was dose-dependently induced in platelets treated with ATO (Fig. 2B). Taken together, these data suggest that ATO induces apoptotic cascades leading to platelet apoptosis.

### ATO Dose-dependently Induces Platelet PS Exposure

**Figure 3:**
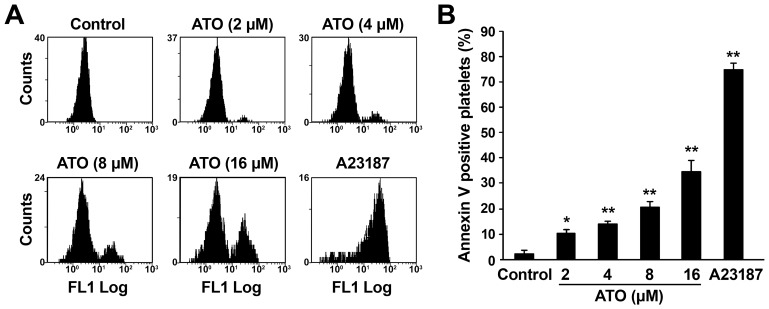
ATO dose-dependently induces PS exposure in platelets. Platelets were pre-treated with 2, 4, 8, 16 μM of ATO or vehicle at 37°C for 5 hrs. (A) Annexin V binding buffer was mixed with the pre-treated platelets and FITC-annexin V at a ratio of 50: 10: 1. The samples were gently mixed and incubated at RT for 15 min in the dark, then analyzed by flow cytometry. (B) Quantitation data from 3 separate experiments with different donors are illustrated (mean ± SD). **P<0.01, *P<0.05 compared with vehicle controls.

PS exposure is a typical apoptotic event in nucleated cells [27], however, PS exposure only occurred in apoptotic process in platelets stimulated with relatively strong agonists [19]–[22]. To investigate whether ATO stimulation is strong enough to induce PS exposure during apoptosis, ATO-treated platelets were examined for annexin V binding. As shown in Fig. 3, ATO dose-dependently induced platelet PS exposure, suggesting that ATO is a potent apoptosis inducer.

### ATO does not Induce Platelet Activation

**Figure 4:**
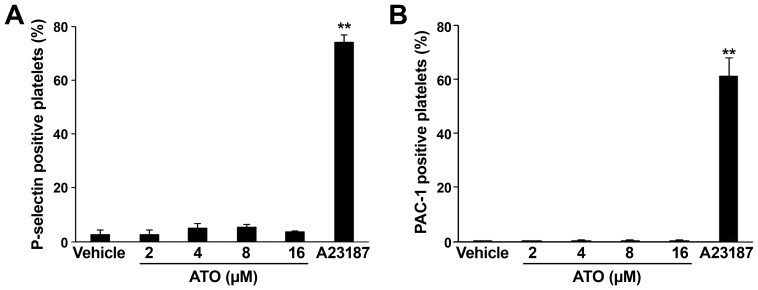
ATO does not incur P-selectin surface expression and PAC-1 binding in platelets. Washed Platelets were treated with different concentrations of ATO (2, 4, 8, 16 μM) or vehicle at 37°C for 5 hrs. (A) Pre-treated platelets were further incubated with SZ51 or mouse IgG at RT for 30 min, and then incubated with FITC-GAM and subjected to flow cytometry analysis. Quantitation data from three separate experiments with different donors are shown as mean ± SD. (B) Pre-treated platelets were further incubated with FITC-labeled soluble PAC-1 at RT for 20 min in the dark, then the treated platelets were fixed with 1% cold paraformaldehyde, further incubated at 4°C in the dark for 30 min and then subjected to flow cytometry detection. Quantitation data from three separate experiments with different donors are shown as mean ± SD (*n* = 3). **P<0.01 compared with vehicle controls.

Platelet surface PS exposure can be caused by both platelet apoptosis and platelet activation [19]–[22]. To exclude the possibility that ATO induces platelet activation, the effects of ATO on platelet activation were investigated. Platelets were incubated with different concentrations of ATO, and then the surface expression of P-selectin was examined by flow cytometry. Compared with vehicle (negative) and A23187 (positive) controls, ATO does not induce obvious surface expression of P-selectin (Fig. 4A). To further clarify whether ATO incurs platelet activation, the activation of integrin αIIbβ3 was detected by FITC-conjugated PAC-1 binding. The result shows that there was no obvious PAC-1 binding in platelets treated with different concentrations of ATO (Fig. 4B). Taken together, these data suggest that ATO does not incur platelet activation.

### ATO Induces Platelet JNK Activation

**Figure 5:**
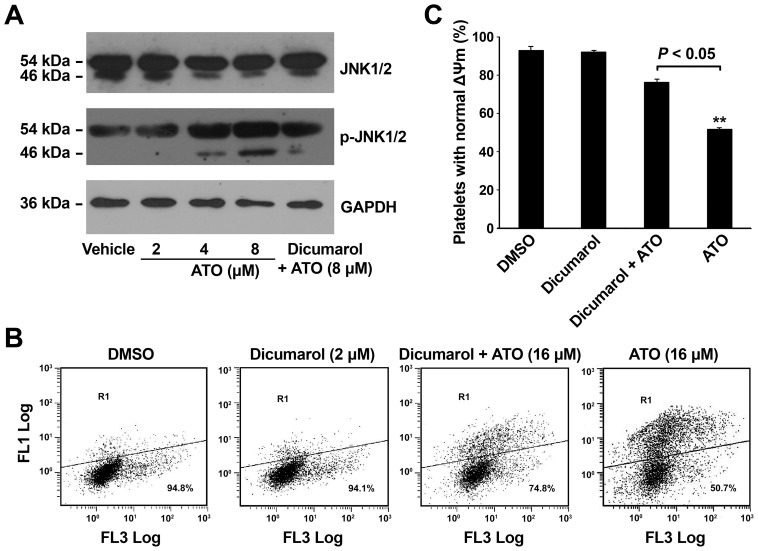
ATO induces JNK activation and dicumarol reduces ATO induced ΔΨm depolarization in platelets. (A) Washed platelets were pre-treated with or without dicumaol (2 μM) or vehicle at 37°C for 15 min, then further incubated with various concentration of ATO at 37°C for 5 hrs. The treated platelets were subjected to Western blot analysis using anti-nonphosphorylated JNK1 and JNK2 antibody (anti-JNK 1/2) and anti-phosphorylated JNK1 and JNK2 antibody (anti-p-JNK 1/2). GAPDH levels demonstrate similar loading. Results are representative of 3 separate experiments with different donors. (B) Washed platelets were pre-treated with or without dicumarol (2 μM) or vehicle at 37°C for 15 min, then further incubated with or without ATO at 37°C for 5 hrs. JC-1 was added into the treated platelets to a final concentration of 5 μg/mL and then incubated at 37°C in the dark for 20 min. The treated samples were detected using flow cytometry. Dots inside R1 are platelets out of the main population bearing ΔΨm depolarization. (C) Quantitation data from 3 separate experiments with different donors are illustrated (mean ± SD). **P<0.01 compared with vehicle controls.

ATO-induced JNK activation was crucial and required for subsequent apoptotic events, such as caspase activation and mitochondrial damage in nucleated cells [17], [18]. Moreover, JNK activation was the earliest response to ATO, preceding and determining reactive oxygen species production [18]. In order to explore the mechanism of ATO-induced platelets apoptosis, we investigate whether ATO incurs JNK activation in platelets. There are three JNK kinases encoded by related genes: JNK1, JNK2, and JNK3. JNK1 and JNK2 are expressed widely in different tissues, whereas JNK3 is mainly expressed in the nervous system [28]. JNK activation is characterized by enhanced phosphorylation of JNK proteins [17], [18]. Thus, ATO-induced JNK activation were examined in platelets stimulated with various concentrations of ATO by the increased phosphorylation of JNK1 (46 kDa) and JNK2 (54 kDa). As illustrated in Fig. 5A, phosphorylated JNK1 and JNK2 were dose-dependently increased by ATO.

To further clarify the effects of ATO on platelet JNK activation, platelets were pre-incubated with dicumarol, the JNK-specific inhibitor, which has been shown to significantly inhibit JNK activation induced by ATO [17], then treated with ATO and subjected to Western blot analysis. The results showed that p-JNK was obviously reduced by dicumarol (Fig. 5A). Taken together, these data indicate that ATO induces platelet JNK activation.

### Inhibition of JNK Attenuates ATO-induced Platelet Apoptosis

It has been clarified that ATO-induced JNK activation is the earliest response preceding apoptotic events in nucleated cells [17], [18]. To further confirm the role of JNK activation in ATO induced platelet apoptosis, platelets were pre-incubated with the JNK specific inhibitor dicumarol, and then incubated with ATO and subjected to ΔΨm analysis. The results showed that dicumarol obviously reduced ATO-induced platelet ΔΨm reduction (Fig. 5B, C), indicating that JNK activation plays a key role in ATO-induced platelet apoptosis.

### ATO Reduces Collagen, ADP, and Thrombin Induced Platelet Aggregation

**Figure 6:**
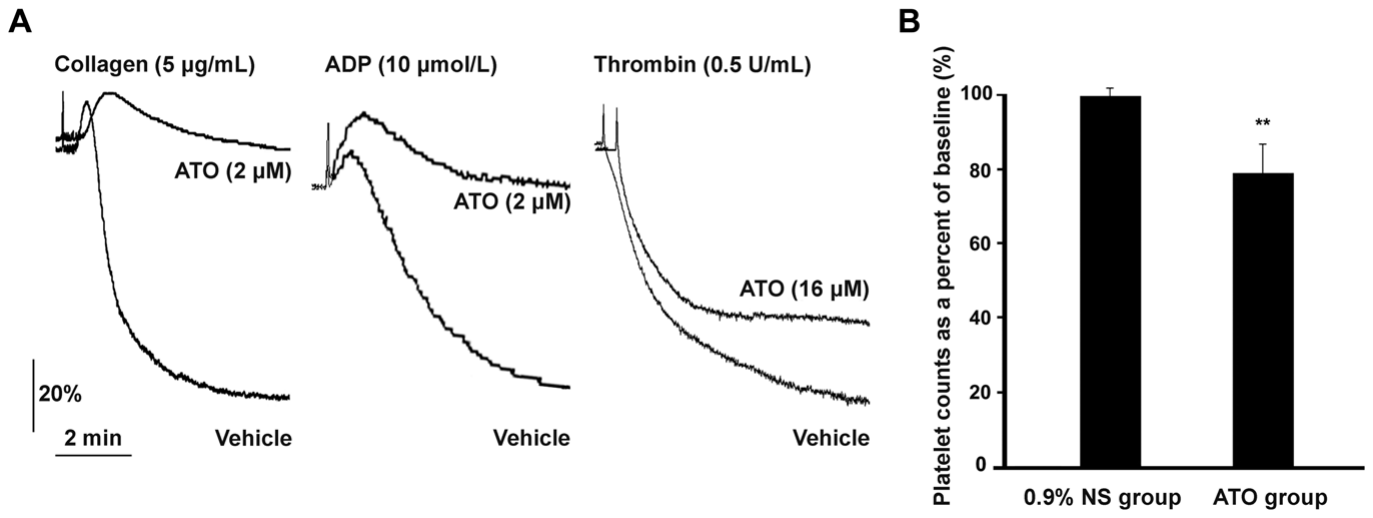
ATO reduces collagen, ADP, and thrombin induced platelet aggregation, and intraperitoneal injection of ATO reduces circulating platelets. (A) PRP was incubated with ATO (2 μM) or vehicle for 1 hr, washed platelets was incubated with ATO (16 μM) or vehicle for 2 hrs. Platelet aggregation assay was performed by addition of collagen (5 μg/mL) or ADP (10 μmol/L) into PRP, or thrombin (0.5 U/mL) into washed platelets under stirring condition, and recorded by a turbidometric platelet aggregometer. Results are representative of 3 separate experiments with different donors. (B) Two groups of mice were intraperitoneally injected with ATO (dissolved in 0.9% NS, 5 mg/kg) or vehicle once a day for 5 continuous days. Blood samples were taken from the mice before injection and 24 hrs after the final injection, and then subjected to platelet count assay using Sysmex KX-21N Blood Cell Analyser. Platelet count (% of baseline) equals platelet count after injection/platelet count before injection ×100%. Quantitation data from 12 mice per group are illustrated as mean ± SD. **P<0.01, compared with vehicle controls.

It has been reported that ATO inhibited collagen, arachidonic acid, thrombin, and U46619 induced human platelet aggregation [29]. In another report, ATO inhibited Sprague-Dawley rat platelet aggregation induced by ADP, arachidonic acid, and epinephrine, however, collagen-induced platelet aggregation was enhanced by ATO [30]. To further clarify the effect of ATO on human platelet aggregation, PRP was incubated with clinical concentration of ATO (2 μM) for 1 hr and then subjected to platelet aggregation assay with collagen and ADP. As shown in Fig. 6A, collagen and ADP induced platelet aggregations were obviously reduced by ATO. Furthermore, thrombin induced platelet aggregation was also slightly reduced in washed platelets incubated with ATO (16 μM) for 2 hrs (Fig 6A).

### ATO Reduces Circulating Platelets

Thrombocytopenia is one of the most frequent hematological side effects during the treatment of ATO in patients with APL and other kinds of malignant diseases [6], [9]–[13]. However, the pathogenesis of ATO-related thrombocytopenia remains unclear. The above data confirm that ATO incurs platelet apoptosis *in vitro*. To further investigate whether ATO reduces circulating platelets *in vivo*, clinical therapeutic dosage of ATO (or vehicle (0.9% NS) control) was intraperitoneally injected into C57 mice (31), and the numbers of circulating platelets were counted. There was no difference in the numbers of circulating platelets between the two groups before the injection of ATO or 0.9% NS (data not shown). However, circulating platelets were moderately but significantly reduced after continuous injection of ATO for 5 days (Fig 6B). These data directly demonstrate that ATO reduces circulating platelets in C57 mice.

## Discussion

ATO has become one of the most effective anticancer drugs, especially in the treatment of APL [1]–[3]. Although it has generally accepted that ATO exerts its anticancer effect by inducing different kinds of malignant cells apoptosis [11]–[15], it still remains unclear whether ATO incurs platelet apoptosis. In the current observation, ATO dose-dependently induces ΔΨm depolarization, up-regulation of Bax and down-regulation of Bcl-2 and Bcl-X_L_, caspase-3 activation, and PS exposure, providing sufficient evidence to indicate that ATO incurs mitochondria-mediated intrinsic platelet apoptosis. In addition, we have tried to explore the signaling cascades leading to ATO-induced platelet apoptosis, and the data indicate JNK activation is involved in the apoptotic process. However, JNK inhibitor did not completely block ATO-induced platelet apoptosis suggesting other signaling pathway might be involved.

ATO induced obvious PS exposure. Since platelet surface PS exposure is a marker of both platelet apoptosis and platelet activation [19]–[22], two typical markers of platelet activation, surface expression of P-selectin and PAC-1 binding, were examined in ATO-treated platelets. The results exclude the possibility that ATO induces platelet activation. It has been reported that ATO inhibited arachidonic acid, thrombin, U46619, and epinephrine induced human platelet aggregation [29], [30]. However, the effect of ATO on collagen-induced platelet aggregation remains conflict [29], [30]. Our data clearly show that ATO reduced collagen, ADP, and thrombin induced platelet aggregation, thus further indicating that ATO impairs platelet functions. We also found relatively higher concentration of ATO was needed to reduce thrombin-induced platelet aggregation, the reason maybe that the washed platelets were desensitized and thrombin is a relatively strong agonist. As for collagen-induced platelet aggregation was enhanced by ATO in Sprague-Dawley rat platelets [30], we think it may because that the reactivity of platelets from the rat is different from that of human platelets. Taken together, these data suggest that ATO-related platelet dysfunction might contribute to hemorrhage in some patients during ATO treatment.

Thrombocytopenia has become one of the most frequent hematological side effects during the treatment of ATO in various kinds of malignancy [6], [9]–[13], particularly in the treatment of APL [7], [9]. Although the most important thing in the treatment of malignant diseases is remission, there are many hemorrhage-related deaths which have been reported during ATO treatment [7], [8]. Thus, the current study is seeking to explore the pathogenesis of ATO remedy-related thrombocytopenia. Clinical pharmacokinetic study of ATO in APL patients indicated that plasma arsenic peak level was from 5.54 μM to 7.30 μM [31]. Upon the evidence that ATO induced platelet apoptosis *in vitro*, ATO was intraperitoneally injected into C57 mice at a dose of 5 mg/kg. According to the previous report, the circulating ATO levels in mice at this dose were similar to that of APL patients treated with ATO [31], [32]. Interestingly, a moderate but significant decrease of circulating platelets occurred after ATO injection. Although it is reasonable to think that the platelet reduction was resulted from apoptosis, there is also the possibility whether ATO incurs bone marrow suppression leading to the decrease of platelet production. Actually, a lot of evidence has confirmed that there is no bone marrow hypoplasia at the dose of ATO from 0.15 mg/kg/d to 0.3 mg/kg/d [6], [32]. On the contrary, ATO exerts a stimulatory action on megakaryocytic differentiation *in vitro* [33], and a group even reported that the numbers of platelet were obviously increased after the treatment with ATO in severe aplastic anemia patients at a dose of 0.15 mg/kg [34]. There is a report that ATO exerts dose-dependent effects on APL cells, under high concentration (1–2 μM), ATO induces apoptosis, under low concentrations (0.1–0.5 μM) and with a longer treatment course, ATO tends to promote differentiation of APL cells [14]. Thus, the conflict on the effects of ATO on platelet count between our model and the severe aplastic anemia patients might result from the different doses of ATO. In addition, as described above, though ATO impaired platelet function, it did not activate platelet. Taken together, these data suggest that ATO-induced platelet apoptosis results in thrombocytopenia *in vivo*.

It is the first time to use an animal model to verify that a compound incurring platelet apoptosis *in vitro* reduces platelet count *in vivo.* However, whether the apoptotic platelets undergo autolysis in circulation or are cleared by reticuloendothelial system still remains to be further investigated.

In conclusion, the data demonstrate that ATO induces caspase-dependent apoptosis via JNK activation in platelets. ATO does not incur platelet activation, whereas, it not only impairs platelet function but also decreases circulating platelets *in vivo*, suggesting the possible pathogenesis of thrombocytopenia in patients treated with ATO.
